# Default Mode Network Connectivity and Social Dysfunction in Major Depressive Disorder

**DOI:** 10.1038/s41598-019-57033-2

**Published:** 2020-01-13

**Authors:** Ilja M. J. Saris, Brenda W. J. H. Penninx, Richard Dinga, Marie-Jose van Tol, Dick J. Veltman, Nic J. A. van der Wee, Moji Aghajani

**Affiliations:** 1 Department of Psychiatry, Amsterdam Neuroscience, Amsterdam UMC, Vrije Universiteit, Amsterdam, The Netherlands; 2 Department of Psychiatry, Leiden University Medical Centre, Leiden, The Netherlands; 3 Leiden Institute for Brain and Cognition, Leiden, The Netherlands; 4 BCN Neuroimaging Center, University Medical Center Groningen, University of Groningen, Groningen, The Netherlands

**Keywords:** Social behaviour, Human behaviour, Depression

## Abstract

Though social functioning is often hampered in Major Depressive Disorder (MDD), we lack a complete and integrated understanding of the underlying neurobiology. Connectional disturbances in the brain’s Default Mode Network (DMN) might be an associated factor, as they could relate to suboptimal social processing. DMN connectional integrity, however, has not been explicitly studied in relation to social dysfunctioning in MDD patients. Applying Independent Component Analysis and Dual Regression on resting-state fMRI data, we explored DMN intrinsic functional connectivity in relation to social dysfunctioning (i.e. composite of loneliness, social disability, small social network) among 74 MDD patients (66.2% female, Mean age = 36.9, *SD* = 11.9). Categorical analyses examined whether DMN connectivity differs between high and low social dysfunctioning MDD groups, dimensional analyses studied linear associations between social dysfunction and DMN connectivity across MDD patients. Threshold-free cluster enhancement (TFCE) with family-wise error (FWE) correction was used for statistical thresholding and multiple comparisons correction (*P* < 0.05). The analyses cautiously linked greater social dysfunctioning among MDD patients to diminished DMN connectivity, specifically within the rostromedial prefrontal cortex and posterior superior frontal gyrus. These preliminary findings pinpoint DMN connectional alterations as potentially germane to social dysfunction in MDD, and may as such improve our understanding of the underlying neurobiology.

## Introduction

Adaptive social functioning is necessary for human survival^1,2^. Regretfully, social behavior is often severely hampered in neuropsychiatric diseases such as Major Depressive Disorder (MDD)^3,4^ with residual dysfunction remaining even after complete remission of depressive symptoms^5,6^. Recent data even suggests that successful MDD remission requires not only a decrease in depressive symptoms but also significant improvements in the social domain^4^. Consonant with this premise, dysfunctions in the social domain are considered an important aspect of MDD^7^ and are according to patients one of the most debilitating consequences of the disorder^8^. Social dysfunction has been studied and established in various ways in MDD patients over the years^7,9^, yet a complete and integrated understanding of the underlying neurobiology is still lacking^3,4^. Knowledge on how major neurobiological systems may contribute to social dysfunction in MDD could allow novel insights into underlying pathomechanisms and aid clinical care.

A neurobiological system potentially relevant to both social (dys)functioning and MDD pathophysiology is the brain’s Default Mode Network (DMN), which has been shown to play a critical role in various aspects of human social behavior^10–14^. The complexity of DMN function and its subsystems is reflected by the broad scope of brain areas involved in the DMN^14–16^. The DMN consists of 2 subsystems and one mediating core system^15^. The core DMN system mainly processes personally relevant, sociocognitive information, with the rostromedial prefrontal cortex (PFC) and posterior cingulate cortex being its key nodes^14,15^. The medial temporal DMN subsystem is associated with recollection of experiences and autobiographical processing, and is comprised of the hippocampal formation, retrosplenial cortex, inferior parietal lobule, and ventromedial PFC^14,15^. The dorsal medial DMN subsystem, on the other hand, is predominantly involved in socially-colored, meta-cognitive processes and mentalizing (i.e., inferences about others’ internal state)^14,15^. The dorsal medial subsystem comprises the temporal poles, lateral temporal cortex, temporoparietal junction, superior frontal gyrus and dorsomedial PFC^14,15^. The different subsystems of the DMN are highly intertwined and this allows for complex interactions and parallel functioning, which is a key ingredient to DMN modulation of intricate human social behaviors^15^. Along the same line, disturbances in one area or subsystem of DMN tend to trigger widespread disruptions across the DMN^14,15^. A whole-network approach of DMN among socially dysfunctional MDD patients, capturing DMN network connectivity in its entirety, thus seems more adequate as an initial, hypothesis-generating step than investigating DMN subsystems.

Alterations in DMN connectional integrity among MDD patients have been described in several overview papers, and putatively linked to deficits in sociocognitive processes that the DMN seems to subserve (e.g., self-referential processing, mentalizing, emotion recognition/resonance)^4,17–19^. The most consistent finding across all studies is altered functional connectivity patterns within prefrontal nodes of the DMN, particularly in rostromedial/ventromedial PFC regions^17,19–23^. Of note, DMN disturbances are also observed in other neuropsychiatric disorders characterized by severe social dysfunctioning, including autism, schizophrenia and social phobia^24–26^, thus further corroborating the importance of DMN to both normal and disturbed social functioning. ﻿As stated by Kaiser *et al*.^18^ in their seminal overview paper, specific patterns of network dysfunction may contribute to core deficits in social, cognitive, and affective functions that could trigger clinical symptoms in neuropsychiatric disorders such as MDD. Hence, a functional network approach towards social functioning in MDD offers the opportunity to study the dynamics of interconnected areas that interact to allow adaptive social behavior.

In summary, social function is often severely impaired in MDD, and alterations in key social processes may be reflected by changes in DMN connectivity. To our knowledge, the intrinsic connectional integrity of DMN has not been studied yet in relation to social dysfunction in MDD. Probing how social dysfunction may both categorically and dimensionally^27–29^ relate to DMN connectivity in MDD could, however, further our understanding of underlying neurobiology and plausibly aid future treatment strategies. This premise is increasingly echoed in the field, and in particular by the Pan-European PRISM study^30^, which upholds that social dysfunction may have a distinct neurobiological signature, be transdiagnostic in nature, and carry clinical/therapeutic relevance. Social functioning, however, is a complex and dynamic process, and often difficult to capture adequately along one specific domain. In order to cover social functioning more broadly and fully, here we assess the cumulative association of three important social dysfunction indices and DMN connectivity within MDD patients. These indices include loneliness, perceived social disability, and small social network; factors not only present among MDD patients in varying levels but also associated with adverse neurobiological changes^11,31–38^. Using this cumulative social dysfunction score, we explored the effect of social dysfunction on DMN whole-network connectivity among MDD patients, both categorically and dimensionally. The categorical analyses examined whether DMN connectivity differs between high and low social dysfunction MDD groups, while the dimensional analysis tested whether a linear association can be found between social dysfunction and DMN connectivity across MDD patients. Post-hoc sensitivity analyses additionally investigated the influence of current comorbid anxiety disorder, antidepressant use, and depression severity on DMN-social dysfunction relationships.

## Results

### Sample characteristics

The mean age of the study sample (*N* = 74) was 36.9 years (*SD* = 11.9) and 66.2% were females (Table 1). MDD patients low in social dysfunction were younger, more often female and had a higher level of education. Whereas depressive symptom severity was slightly higher in the MDD high social dysfunction group, the other clinical psychiatric characteristics (comorbid anxiety disorder, antidepressant use, symptom duration, age of onset) did not differ between groups. Within the total sample, 54.1% had a current comorbid anxiety disorder and a mean IDS score of 21.5 (mild depressive symptomatology). Participants had symptom durations of on average 33.5% of all follow-up months and the mean age of onset was 25.1 years. Antidepressants were used by 31.1% of the participants.

**Table 1:** Sample Characteristics.

Variable	MDD high social dysfunction (N = 37)	MDD low social dysfunction (N = 37)	p-value, effect sizes (Cohen’s *d|Phi)*	MDD entire sample (N = 74)
Age (mean ± SD)	39.8 (11.9)	33.9 (11.3)	0.03; 0.5	36,9 (11,9)
Sex (% female)	51.4%	81.1%	0.01; 0.3	66,2%
Years of education (mean ± SD)	11.4 (2.0)	12.6 (2.9)	0.03; 0.5	12,0 (2,4)
**Number of individuals per scan site**				
- UMCG Groningen	15	8		
- LUMC Leiden	15	18		
- Amsterdam UMC, Amsterdam	7	11		
Comorbid anxiety disorder (%)	64.7%	43.2%	0.06; −0.2	54.1%
Antidepressant use (%)	40.5%	21.6%	0.08; −0.2	31.1%
Depression severity (IDS) (mean ± SD)	26.6 (11.1)	19.7 (10.2)	0.01; 0.6	23.1 (11.2)
Symptom duration (% time with symptoms)	39.7%	27.2%	0.06; 0.5	33.5%
Age of onset (years) (mean ± SD)	25.6 (11.9)	24.7 (10.7)	0.01; 0.01	25.1 (11.3)
Social Dysfunction				
*Standardized/log-transformed (mean ± SD)*				
- Social dysfunction composite ^**^	0.9 (0.2)	−0.02 (0.5)	0,00; 2,9	0.4 (0.6)
- Loneliness^**^	1.0 (0.3)	0.1 (0.7)	0,00; 1,7	0.6 (0.7)
- Perceived social disability^**^	1.1 (0.3)	0.0 (0.8)	0,00; 1,8	0.6 (0.8)
- Small Social Network^**^	0.6 (0.3)	−0.2 (1.0)	0,00; 1,1	0.2 (0.9)
*Raw (mean* ± *SD)*				
- Loneliness^**^	8.7 (2.4)	4.2 (2.6)	0,00; 1,8	6,4 (3,4)
- Perceived social disability^**^	15.7 (3.4)	9.8 (3.7)	0,00; 1,7	12,8 (4,5)
- Small Social Network^**^	5.1 (0.5)	3.9 (1.0)	0,00; 1.5	4.5 (1.0)

Chi-square tests were employed for categorical variables, and independent sample t-test for continuous variables. Effect sizes for continuous data was calculated using Cohen’s d, for dichotomous data phi coefficient.

Higher scores on social dysfunction measures denote more subjectively experienced social dysfunction. A higher social dysfunction composite score thus indicates more severe social dysfunction (more loneliness, higher perceived social disability, smaller social network). IDS = Inventory of depressive symptomatology.

* = *P* < 0.05; **P < 0.001; ns = not significant at *P* < 0.05

Note: Data on depressive duration missing in 1 MDD patient with low social dysfunction.

Data on depressive severity missing in 2 MDD patients: 1 high and 1 low on social dysfunction.

### Functional connectivity analysis

**Figure 1:**
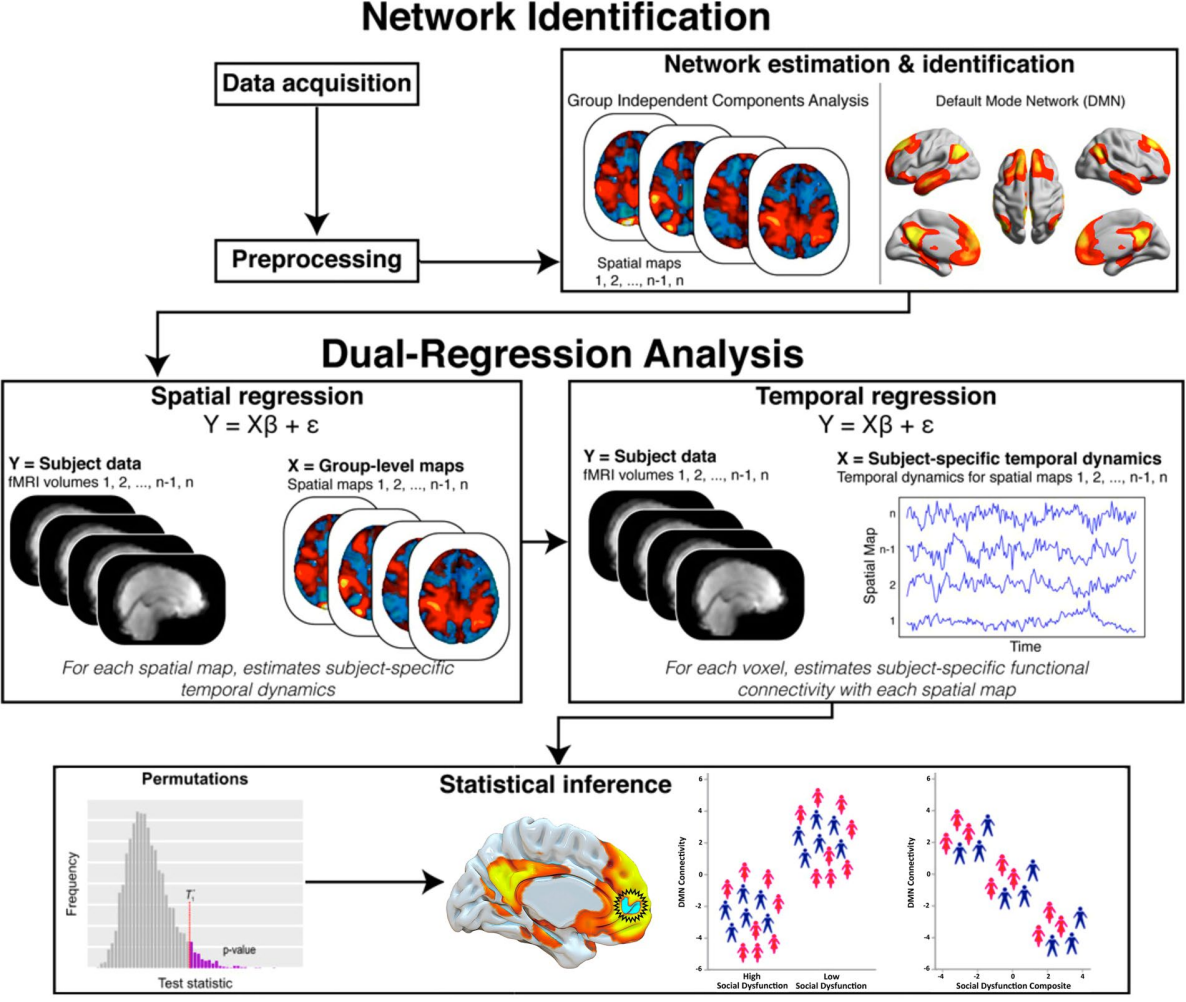
Functional connectivity analyses of the Default Mode Network (DMN). Collected resting-state fMRI data were first extensively preprocessed and cleaned^67^. Data from all participants was next concatenated across time and submitted to a probabilistic group independent component analysis (ICA) using MELODIC. The group ICA produced a set of 20 independent spatial maps/components (i.e., functional networks). The set of spatial maps generated by MELODIC was then used to generate subject-specific versions of these spatial maps, and associated time courses, using Dual Regression. That is, for each subject, the group-average set of spatial maps was regressed (as spatial regressors in multiple regression) onto the subject’s 4D space-time dataset. This resulted in a set of subject-specific time series, one per group-level spatial map. Next, these time series were regressed (as temporal regressors, again using multiple regression) against the same 4D dataset, resulting in a set of subject-specific spatial maps, one per group-level spatial map. Our component of interest (i.e., DMN) was then selected based on spatial similarity to functional networks described in prior seminal papers on DMN connectivity and architecture. Finally, permutation testing (*N* = 5000) was used to probe the association between DMN connectivity and social dysfunction, both categorically and dimensionally, while correcting for age, sex, education, and scanner location. Results were adjusted for multiple comparisons using Threshold-Free Cluster Enhancement with Family-Wise Error correction at *P* < 0.05. Adapted and reprinted with permission from Wiley Periodicals, Inc.: Human Brain Mapping^78^

**Figure 2:**
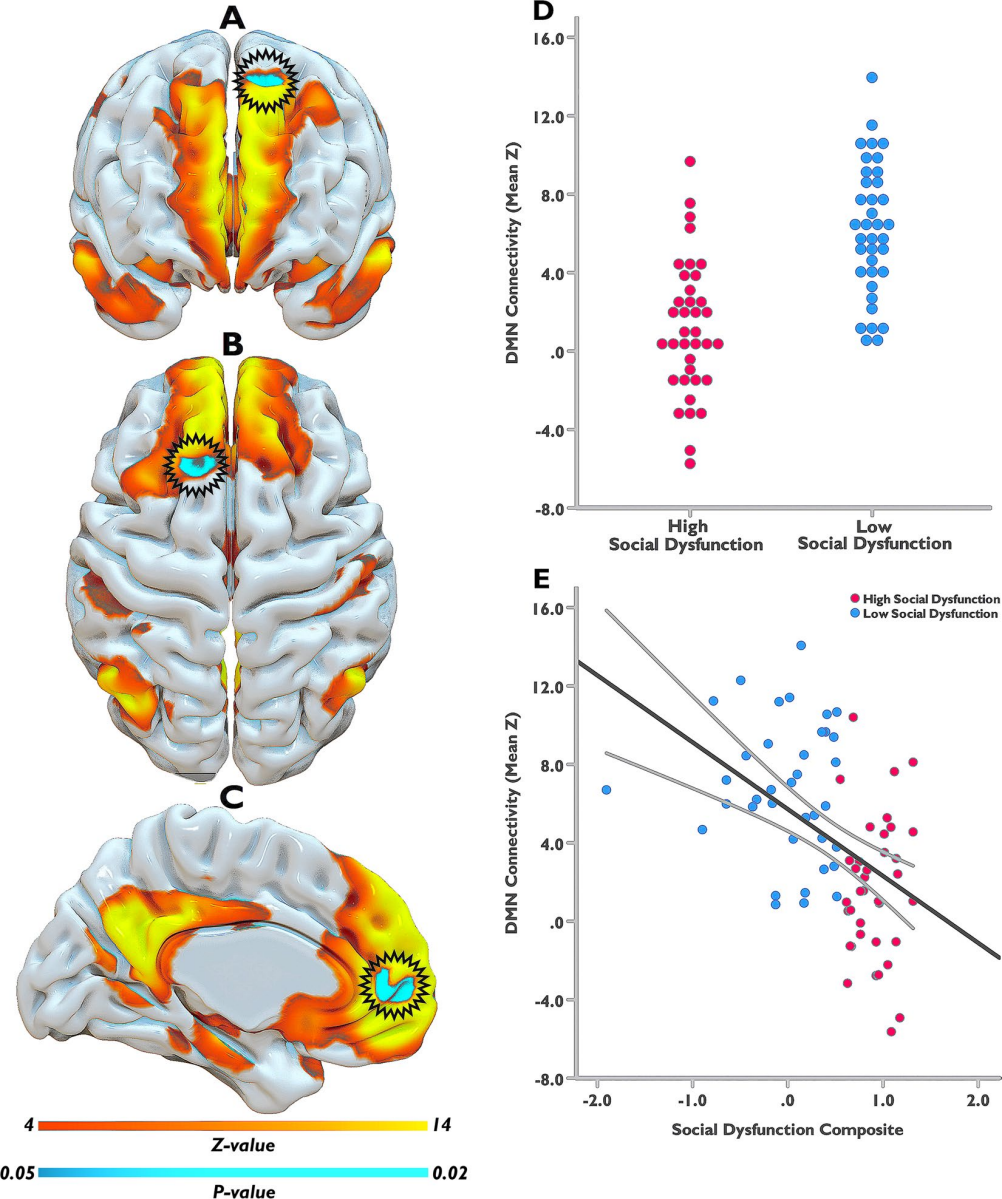
DMN connectivity and social dysfunction in MDD patients. The left panel depicts anterior (**A**), superior (**B**), and medial (**C**) views of the DMN (yellow-orange), along with its rmPFC and pSFG subregions (blue) that showed diminished connectivity in MDD patients with high vs. those with low social dysfunction (*TFCE* & *FWE* corrected, *P* < 0.05). The rmPFC effect site is depicted in figure (**C**) and the pSFG site in figures (**A,B**), with black edged circles marking the effect sites for better visibility. The yellow-orange scalar bar represents connectivity strengths (*Z-value*) within DMN, while the blue scalar bar reflects significance level of between-group differences in DMN connectivity (*P-value*). The distribution plot (middle panel, D) provides a quantitative visualization of this categorical between-groups effect, wherein mean connectivity estimates from the DMN effect sites (y axis) are plotted for each group separately (x axis). Exploratory dimensional analysis focusing on effect sites from the categorical analysis (i.e., parts of the rmPFC and pSFG), revealed the same pattern of diminished DMN connectivity as a function of higher social dysfunction levels across participants (*TFCE* & *FWE* corrected, *P* < 0.05). The scatter plot (middle panel, E) provides a quantitative visualization of this effect, wherein mean connectivity estimates from the DMN effect sites (y axis) are plotted against social dysfunction composite scores (x axis). The black line depicts the slope of the association, with the grey bands indicating the 95% confidence interval of the slope. DMN = Default Mode Network; MDD = Major Depressive Disorder; rmPFC = Rostromedial Prefrontal Cortex; pSFG = Posterior Superior Frontal gyrus; TFCE = Threshold Free Cluster Enhancement; FWE = Family-Wise Error.

Twenty functional networks were generated during the probabilistic Independent Component Analysis (ICA) and entered into Dual Regression, with the DMN network being selected for further analysis (Fig. 1 and 2). The DMN has been consistently found across subjects and over time using the same methods as applied here^39,40^, with DMN architecture also emerging among all of our participants. Using the composite social dysfunction score, we next examined the association of social dysfunction and DMN connectivity in MDD patients, both categorically and dimensionally. Whole-DMN *categorical* analyses revealed diminished DMN connectivity in high social dysfunction MDD patients, specifically within the rostromedial prefrontal cortex (rmPFC) and posterior superior frontal gyrus (pSFG) subsections of the DMN (*TFCE* & *FWE* corrected, *P* < 0.05) (Fig. 2). Our whole-DMN dimensional analyses similarly suggested a pattern of reduced DMN connectivity as a function of more social dysfunction, though this effect was not statistically significant (*TFCE & FWE* corrected, *P* = 0.33). Exploratory analyses revealed that at a more lenient threshold (*P* < 0.001, uncorrected), the same pattern of diminished DMN connectivity within the left rmPFC and pSFG could be observed, with an average correlation *r* = −0.43, *P*-uncorrected <0.001. When we moreover reran the *dimensional analyses* focusing specifically on effect sites from the *categorical analysis* (i.e., parts of the left rmPFC and pSFG), we similarly found an association between diminished DMN connectivity and higher social dysfunction levels across participants (*TFCE* & *FWE* corrected, *P* < 0.05) with an average correlation *r* = −0.58, *P*-corrected < 0.05 (see Fig. 2). Taken as a whole, our analyses cautiously link more severe social dysfunctioning among MDD patients to diminished DMN connectivity, in particular when comparing low and high social dysfunctioning MDD patients.

### Sensitivity analyses

The post-hoc sensitivity analyses revealed that between-group differences in DMN connectivity in high vs. low social dysfunctioning MDD groups remained significant, while excluding MDD patients with current comorbid anxiety disorders (*N* = 40 excluded) (*F*(1,27) = 21.98, *P* < 0.05) or using antidepressants (*N* = 23 excluded) (*F*(1,44) = 66.84, *P* < 0.05). Including all patients and covarying for comorbidity, antidepressant use, and depression severity, on top of age, sex, education, and scanner location, also did not affect the documented between-group DMN effects (*F*(1,64) = 48.26, *P* < 0.05).

### Composite score vs. individual social dysfunction indices

We opted to use a cumulative measure of social dysfunctioning and examine its association with DMN connectivity among MDD patients. This cumulative measure was generated by combining three separate questionnaires that were most affected in MDD patients with social dysfunction, as reflected in their medium to large effect sizes (ranging from 0.54–1.19). In addition, for these three questionnaires, there is considerable evidence of their impact on neurobiological indicators^11,32–37,41–43^. Lastly, these indicators are also employed in the Pan-European PRISM study on the neurobiology of social dysfunction^30^. Each questionnaire assesses a different domain of social dysfunction: loneliness, perceived social disability, and a small social network. This resulted in a social dysfunction composite index that (1) captures multiple domains of social dysfunctioning at once and more fully than each individual measure separately, (2) makes multiple testing of brain-behavior relations for each individual measure redundant, (3) and allows insight into the *cumulative association* of social dysfunction on brain network connectivity. The fact that the three separate questionnaires were also highly correlated (*r* = 0.40–0.50, *P’s* < 0.01), and thus prone to multicollinearity, further justifies the use of the composite score rather than individual questionnaires. Moreover, when we reran our connectivity analyses using the total sum scores of each social dysfunction questionnaire (both separately and in one model), no significant DMN effects emerged, and none of the questionnaires’ total sum score was predictive of DMN connectivity strength in the effect sites documented here (*TFCE* & *FWE* corrected, *P’s* > 0.20). These findings thus cautiously hint that the cumulative social dysfunction index is ostensibly better able to pick up subtle brain-behavior relations, at least in this specific dataset, which echoes to some extent the current understanding on the topic^4^.

## Discussion

The current study explored the relation between social dysfunctioning (operationalized as a composite of loneliness, perceived disability and small social network) and DMN whole-network connectivity among MDD patients. The analyses cautiously linked greater social dysfunctioning among MDD patients to diminished DMN connectivity, specifically within the rmPFC and pSFG. These preliminary findings pinpoint DMN connectional alterations as potentially germane to social dysfunction in MDD, and may as such improve our understanding of the underlying neurobiology.

### DMN connectivity and social dysfunction

One of the key findings of this study is diminished connectional integrity of the DMN within its rmPFC subregion among MDD patients with more severe social dysfunction. This finding builds upon prior research suggesting that DMN connectional integrity is not only indispensable to adaptive human social functioning^10,11,15^, but also to positive social interaction^43^. It furthermore echoes findings in other neuropsychiatric disorders also characterized by severe social deficits (i.e., schizophrenia, autism), wherein diminished DMN connectivity with its rmPFC node similarly relates to more severe social dysfunctioning^12,44^. Of note, disruptions across multiple brain networks with the rmPFC as the core region are reported in MDD patients, and tentatively implicated as a key pathological feature of the disorder^22,23^. The rmPFC region is a key node of the DMN core system and mainly supports self-relevant sociocognitive and socioaffective processes^14,15^. The rmPFC, as part of the DMN, is for instance activated when one’s memory is employed to construct future social scenes^45^, and also supports emotion regulation by drawing on past experiences^10^. The rmPFC is also implicated in the so-called “extended social affective default network”, which supposedly governs various aspects of higher-order socioaffective information processing^46^. The rmPFC as part of the DMN core system is also crucially involved in coupling between DMN subsystems, which allows for complex interactions and parallel functioning^15^. Connectional disturbances in the rmPFC part of the DMN may therefore not only upset functions tightly coupled to this subregion, but also prompt disruptions across DMN subsystems and their associated functions. This certainly fits the behavioral profile of most MDD patients, wherein a host of social dysfunctions tend to surface, ranging from biased self-related processing and social cognition to impaired interpersonal function^3,4^. These social deficits moreover contribute to greater MDD severity^3,4^, thus further highlighting the relevance of maladaptive social processing to MDD clinical presentation. Taken as a whole, our finding seems to suggest that diminished DMN connectivity, specifically within its rmPFC subregion, may carry relevance for a wide range of social deficits among more socially dysfunctional MDD patients. Future studies are warranted though to further explore and validate our tentative finding and interpretations, given the complexity of the DMN system and its modulation of intricate human social behavior.

We also found diminished DMN connectivity within the pSFG subregion among the more socially dysfunctional MDD patients. The pSFG is a posterosuperior PFC region that borders the precentral gyrus, and is bounded laterally by the superior frontal and cingulate sulci^47^. The pSFG is reckoned as a node within the dorsal medial DMN subsystem, and within this role supportive of interpersonal sociocognitive processes such as mentalizing and theory of mind (ability to understand others’ intentions/emotions/beliefs/desires)^14,15,48^. One may thus speculate that adverse connectional changes in this specific DMN subregion as documented here, could reflect biased mentalizing and theory of mind processes critical to adaptive social function. Depressed patients do in some cases indeed show deficits in these sociocognitive processes^3,4,49,50^, which apparently are to some extent driven by functional anomalies in brain regions that partly fall within the dorsal medial DMN subsystem^3,4^. Of note, more severe mentalizing and theory of mind deficits seemingly also predict increased MDD severity^3,4,49^, which again underscores the importance of impaired social functioning to less favorable MDD clinical presentation. It is interesting that mentalizing and theory of mind deficits in other neuropsychiatric disorders also seem to coincide with altered DMN connectivity with its pSFG subregion^12,51,52^. In sum, our finding may cautiously link altered pSFG connectivity within the DMN to suboptimal interpersonal and social interactive processing in more socially dysfunctional MDD patients. Further investigation and future replication of our finding is warranted though, as within the context of DMN, the contributions of pSFG to (mal)adaptive social processing are still understudied in MDD.

### Categorical versus dimensional approach

Social dysfunction and MDD are two intertwined and extremely complex phenomena that seem notoriously difficult to capture along one dimension or methodology^4^. Solely a categorical or dimensional examination of these two intertwined phenomena would likely lead to a fractured understanding of them and cause loss of information. Following this perspective and consonant with an increasing number of recent studies^27–30,53^, both categorical and dimensional analyses were performed to study the association between social dysfunctioning and DMN connectivity among MDD patients. The dimensional analysis tested whether a linear association could be found between individual participant’s composite score and DMN connectivity across participants (i.e., significant slope across group), while the categorical analyses explored whether DMN connectivity differed between the high and low social dysfunction groups (i.e., different slopes for each group). The categorical analyses revealed diminished DMN connectivity among MDD patients with more severe social dysfunctioning. Whole-DMN dimensional analyses similarly revealed a pattern for reduced DMN connectivity as a function of more social dysfunctioning, though this effect did not pass statistical significance. Exploratory dimensional analysis did show that that these patterns more prominently echoed that of the categorical analysis, when adopting a more lenient threshold (*P* < 0.001, uncorrected), or utilizing a region of interest approach. The exploratory nature of these post-hoc analyses, however, does warrant cautious interpretation, as they mainly served to aid transparency and completeness. The distribution of data and amount of variance across participants vs. within groups, the possibility of ceiling effects, and differences in statistical power may explain the subtle differences in outcomes of the categorical vs. dimensional analyses. In addition, one should consider the possibility that contrasting the extremes (as in categories) may better pick up subtle brain-behavior effects, then when enforcing a linear trend that in reality is subthreshold or perhaps not linear in nature. Yet, taken as a whole, both approaches seem to converge on the same pattern by cautiously linking more severe social dysfunctioning among MDD patients to diminished DMN connectivity. Nonetheless, the preliminary nature of the findings and interpretations do necessitate replication and further exploration to fully appreciate their relevance.

### Limitations and strengths

The cross-sectional and exploratory nature of this study does not allow for firm causal inferences, and longitudinal research in preferably larger samples is warranted to tackle this limitation. The current study employed a composite index of social dysfunction among MDD patients, which notwithstandingly has its merits as mentioned earlier, but in essence remains a subjective proxy for social disability. Social processing and functioning are multifaceted and complex phenomena, which are hard, if not impossible, to capture and reduce to a numerical value. A more in-depth examination of the composition of social networks or the nature of perceived social disability and loneliness are promising avenues for future research. In the end, the dissection of a complex phenotype such as social dysfunction requires the assessment of as much as possible putative contributors^4^. Although the used questionnaires are validated and specifically developed to study different aspects of social dysfunctioning, a more objective approach of social dysfunctioning would be valuable in complementing subjective self-assessments. However, the NESDA cohort study, from which we include a subset of participants, simply lacks more objective measures. The use of more objective measures is therefore beyond the scope of this paper. Validated questionnaires specifically developed to study different aspects of social (dys)functioning were used instead. This study moreover has a within-patient design, with all participants with depression likely experiencing some degree of low confidence within the social domain, as this is a disease-inherent feature. This lack of social confidence, however, should not be seen as an additional source of bias in their self-reported social (dys)functioning. The current study and its findings should thus ideally serve as a point of departure or source of hypothesis generation for more in-depth examination of social dysfunction and its biobehavioral underpinnings in the future. We additionally did not include healthy controls in the analyses, as the main objective was to probe the association between social dysfunction and DMN connectivity specifically and exclusively in MDD patients. While some in the field may deem this a potential limitation, an increasing number of seminal studies on MDD neurobiology are employing this within-patient methodology (e.g.^54,55^,), for it may aid the interpretability of findings. This is especially true in situations wherein explanatory and criterion variables of interest both tend to systematically differ between MDD patients and healthy participants (e.g., differences in general neurobiology, range of social dysfunction, clinical and sociodemographic characteristics). Simply correcting for these factors does not fully eliminate their confounding impact, thus rendering the interpretation of findings more arduous. Moreover, the topological architecture of the DMN can be reliably and consistently represented across populations^40,56^, making the inclusion of healthy controls for the current investigation not a prerequisite. In addition to above-mentioned limitations, it should be noted that the NESDA study excludes patients using non-SSRI antidepressants, which may introduce a selection bias and plausibly mitigate the generalizability of the findings.

Notwithstanding these limitations, our study also has several strengths worth mentioning. It is one of the first studies that explicitly aimed to unravel the neurobiological underpinnings of social dysfunction in MDD. This is of relevance, as social dysfunction has been studied and established in various ways in MDD^7,9^, though a complete and integrated understanding of the underlying neurobiology is still lacking^3^. The sample is moreover very well described and rather homogeneous in terms of clinical presentation, with the high and low social dysfunction groups being not much different on key clinical parameters. We also corrected for relevant clinical and sociodemographic factors, which collectively aid the reliability of the study findings.

## Conclusions

In summary, our preliminary findings cautiously link greater social dysfunctioning among MDD patients to diminished DMN connectivity, specifically within its rmPFC and pSFG subregions. The findings seem to provide relevant, yet preliminary, clues on the neurobiology underlying social dysfunction in MDD, by highlighting DMN connectional disturbances as a potentially important factor. These initial exploratory findings should be further explored and validated, ideally through multimodal examination of DMN connectivity and complex network analyses (e.g., graph theory), to attain a more fine-grained representation of DMN and its network dynamics. The current findings could plausibly serve as a point of departure or source of hypothesis generation for these future endeavors.

## Methods

### Participants

Participants were recruited from the longitudinal, naturalistic Netherlands Study of Depression and Anxiety (NESDA^57^). The study protocol for NESDA was carried out in accordance with guidelines approved by the Ethical Review Board of the VU University Medical Centre and by local review boards at each participating centre (University Medical Center Groningen (UMCG), Leiden University Medical Center (LUMC)). Informed written consent was given by all participants. DSM-IV diagnoses of current (6-month recency) MDD were established using the Composite International Diagnostic Interview lifetime version 2.1. Exclusion criteria for MDD patients within the NESDA-MRI study were the presence of Axis I disorders other than depressive or anxiety disorders (i.e., panic, social anxiety and/or generalized anxiety disorder), use of psychotropic medication other than a stable use of selective serotonin reuptake inhibitors or infrequent benzodiazepine use, presence or history of major internal or neurological disorder, dependency or recent abuse (past year) of alcohol or drugs, hypertension, presence of MRI-contraindications and not being fluent in Dutch language. MDD patients were recruited through general practitioners, primary care, and specialized mental care institutions. Resting-state fMRI data were available for 120 participants with depression. Participants were excluded if their fMRI images were of substandard quality (e.g., due to movements or technical issues, N = 24) or data were missing on social dysfunctioning questionnaires (N = 22). We included 74 individuals with a 6-month DSM-IV diagnosis of MDD (mean age = 36.9, *SD* = 11.9; 66.2% female).

### Social dysfunction

To cover social dysfunctioning a social composite score was calculated using three validated (subscales of) questionnaires that probed loneliness, perceived social disability, and small social network size. These three proxy indicators are moderately correlated with each other (*r = *0.40–0.50, *P’s* < 0.01) and have been shown predictive of social dysfunctioning and adverse neurobiological changes^11,32–37,41–43^. These three indicators of social dysfunctioning moreover emerged as being prominently affected in MDD patients as compared to healthy controls in a separate study by our group (effect sizes ranging from 0.54 to 1.19^38^), and also are employed in the Pan-European PRISM study on the neurobiology of social dysfunction^30^. Subjective feelings of loneliness were measured using the loneliness questionnaire^58^, which consists of 11 items that are scored on a 3-point Likert scale. Perceived social disability, or difficulties in making new or maintaining friendships, was measured using the social interaction subscale domain of the World Health Organization Disability Assessment Schedule (WHO-DAS)^59,60^, which consists of 5 items that are scored on 5-point Likert scale. Social network size was assessed using the close person inventory^61,62^, wherein the number of adults with whom the participant has regular and important contact with is scored on a 6-point ordinal scale (number of individuals in a network: >20, 16–20, 11–15, 6–10, 2–5, 0–1). The social network size scores were reversed, so that in line with the other two questionnaires higher scores would denote more social dysfunction, hence allowing for a more intuitive and reliable composite score. In line with prior work^63^, this composite score was calculated by first log transforming and standardizing the individual questionnaire scores, subsequently summing them up, and then dividing the sum by three. A higher composite score thus indicates more social dysfunction (more loneliness, higher perceived social disability, smaller social network). The correlations between this composite score and the individual questionnaires were all above *r = *0.73 (*P’s* < 0.001). This social dysfunction composite score thus captures multiple domains of social dysfunctioning at once and more fully, makes multiple testing of each individual measure redundant, and allows insight into the cumulative effect of social dysfunction on brain network connectivity.

### MRI data acquisition

Imaging data were acquired using Philips 3 T MR- systems (Best, the Netherlands) located at the LUMC, AMC, and UMCG, equipped with a SENSE-8 (LUMC and UMCG) and a SENSE-6 (AMC) channel head coil respectively. Resting-state fMRI (RS-fMRI) data were acquired using a T2-weighted gradient echo echo-planar imaging with the following scan parameters in Amsterdam and Leiden: 200 whole-brain volumes; repetition time (TR) 2300 ms; echo time 30 ms; flip angle 801; 35 transverse slices; no slice gap; field of view 220 × 220 mm; in-plane voxel size 2.3 × 2.3 mm; slice thickness 3 mm; duration 7.51 min. Parameters in Groningen were identical, apart from: echo time 28 ms; 39 transverse slices; in-plane voxel size 3.45 × 3.45 mm. A sagittal 3-dimensional gradient-echo T1-weighted image was acquired for registration purposes and gray matter analysis with the following scan parameters: repetition time 9 ms; echo time 3.5 ms; flip angle 801; 170 sagittal slices; no slice gap; field of view 256 × 256 mm; 1 mm isotropic voxels; duration 4.5 min. In the darkened MRI room participants were instructed to lie still with their eyes closed and not to fall asleep. Participants confirmed wakefulness after the scanning session. No abnormalities were found upon inspection of the subjects’ structural images by a neuroradiologist.

### MRI data preprocessing

The RS-fMRI imaging data was preprocessed and analyzed using sing FMRIB Software Library (FSL) version 5.0.10 and included removing of scanner, (micro)motion, and physiological artefacts using a combination of FSL FIX^64^, ICA-AROMA^65^, motion correction (realignment) using McFLIRT^66^, grand mean scaling, spatial smoothing with 6 mm Gaussian kernel, high pass filtering (Gaussian-weighted least-squares straight line fitting with a 0.01 Hz cut-off) and is described in depth elsewhere^67^. Additional nuisance signal regression was performed according to pipeline recommended in (50) and consisted of regressing mean signals from the cerebrospinal fluid (CSF) and white matter (WM). CSF and WM masks were obtained by multiplying subject-specific T1 segmentations obtained using FSL’s FAST^68^ with the MNI152-based CSF and WM anatomical priors provided as part of FSL and thresholded with a 0.95 threshold. The resulting RS-fMRI images were registered to Montreal Neurological Institute (MNI) space using transformation matrices obtained from the first co-registration of functional images to T1 image using the FLIRT boundary based registration tool^69^ and registering the T1 images to MNI template brain using FMRIB’s linear image registration tool (FLIRT)^70^. Participants were excluded if head movement was above 2.5 mm | 0.4 rad, or if functional images were of insufficient quality.

### Functional connectivity analysis

Figure 1 depicts the analytical pipeline employed in this study, which we will further outline in the following paragraphs. Functional connectivity analysis was carried out using probabilistic Independent Component Analysis (ICA^71^;), as implemented in FSL’s Multivariate Exploratory Linear Decomposition into Independent Components tool (MELODIC). Default group ICA processing steps were applied to the individual preprocessed and normalized data sets: masking of non-brain voxels, voxel-wise de-meaning of the data, and normalization of the voxel-wise variance based on all data sets. Subsequently, the preprocessed data were concatenated in time to create a single 4D data set that was then projected into a 20-dimensional subspace using principal component analysis. The observations were decomposed into 20 sets of independent vectors that describe signal variation across the temporal (time courses) and spatial (maps) domains by optimizing for non-Gaussian spatial source distributions using a fixed-point iteration technique. We chose to use 20 independent components to reach the same balance between the amount of clustering and splitting as previous studies applying the same techniques and capture the complete DMN^40,56^. In short, probabilistic ICA within MELODIC thus uses all the data available within the fMRI dataset to decompose the entire temporal fMRI dataset into independent spatial components, which relate to intrinsically connected functional brain networks. The set of spatial maps/components generated by MELODIC was used to generate subject-specific versions of the spatial maps, and associated time courses, using Dual Regression^72^. That is, for each subject, the group-average set of spatial maps was regressed (as spatial regressors in multiple regression) onto the subject’s 4D space-time dataset. This resulted in a set of subject-specific time series, one per group-level spatial map. Next, these time series were regressed (as temporal regressors, again using multiple regression) against the same 4D dataset, resulting in a set of subject-specific spatial maps, one per group-level spatial map.

Our component of interest (i.e., DMN; Fig. 1) was then selected based on spatial similarity to functional networks described in prior seminal papers on DMN large-scale connectivity and architecture (e.g.^40,72^,). This component, reflecting the DMN, included the vmPFC, posterior cingulate, retrosplenial cortex, inferior parietal lobule, lateral temporal cortex, and dmPFC. The composite social dysfunction score was used in subsequent statistical inferences to assess the relation between social dysfunction and DMN connectivity in MDD patients, both categorically and dimensionally. In the categorical analyses, MDD patients were divided into a high and a low social dysfunction group based on the group median of the social dysfunction composite (Median = 0.44), and the analyses examined whether the association between composite and DMN connectivity differed between the two groups. The dimensional analysis included all MDD patients in one large group and assessed whether across participants a linear association could be found between individual participant’s composite score and DMN connectivity.

All statistical analyses were performed using FSL’s non-parametric, permutation-based Randomise tool^73^, which included 5000 random permutations to build up the null distribution of the cluster size statistic while testing our contrasts of interest in the categorical and dimensional analyses. Four nuisance regressors (all demeaned across participants) describing age, sex, education, and scanner location were added to the model. Statistical maps were thresholded using Threshold-Free Cluster Enhancement (TFCE^74^,) with family-wise error (FWE) correction at *P* < 0.05 to control for multiple comparisons. TFCE is currently one of the most robust methods for finding significant “clusters” in voxelwise MRI data, without having to define clusters in a binary^74,75^. Cluster-like structures are enhanced but the image remains fundamentally voxelwise^74^. The control of multiple comparisons across relevant voxels was achieved through sequential/serial FWE-correction^74^ with α = 0.05, meaning the chance of false positives occurring over the entire voxel space is no more than 5%.

### Sensitivity analyses

Similar to prior work^76,77^, we performed post-hoc sensitivity analyses to examine the association between current comorbid anxiety disorders and antidepressant use and DMN connectivity. Using individual participants’ connectivity strength level (i.e., mean *Z*-scores) within DMN regions of significant effect, analyses of variance (ANOVA’s) were conducted to compare MDD patients with high versus low social dysfunction, excluding either those with a comorbid disorder or those using antidepressants. Finally, we explored whether covarying for comorbidity and antidepressant use, on top of age, sex, education, and scanner location, would affect any of the findings. All analyses were done using SPSS version 22.0 (SPSS Inc, Chicago, Illinois).

## Data Availability

The data that support the findings of this study are available from www.nesda.nl but restrictions apply to the availability of these data, which were used under license for the current study, and so are not publicly available. Data are however available from the authors upon reasonable request from www.nesda.nl.
